# Musicians have better memory than nonmusicians: A meta-analysis

**DOI:** 10.1371/journal.pone.0186773

**Published:** 2017-10-19

**Authors:** Francesca Talamini, Gianmarco Altoè, Barbara Carretti, Massimo Grassi

**Affiliations:** 1 Department of General Psychology, University of Padua, Padua, Italy; 2 Department of Developmental Psychology and Socialization, University of Padua, Padua, Italy; University of Zurich, SWITZERLAND

## Abstract

**Background:**

Several studies have found that musicians perform better than nonmusicians in memory tasks, but this is not always the case, and the strength of this apparent advantage is unknown. Here, we conducted a meta-analysis with the aim of clarifying whether musicians perform better than nonmusicians in memory tasks.

**Methods:**

Education Source; PEP (WEB)—Psychoanalytic Electronic Publishing; Psychology and Behavioral Science (EBSCO); PsycINFO (Ovid); PubMed; ScienceDirect—AllBooks Content (Elsevier API); SCOPUS (Elsevier API); SocINDEX with Full Text (EBSCO) and Google Scholar were searched for eligible studies. The selected studies involved two groups of participants: young adult musicians and nonmusicians. All the studies included memory tasks (loading long-term, short-term or working memory) that contained tonal, verbal or visuospatial stimuli. Three meta-analyses were run separately for long-term memory, short-term memory and working memory.

**Results:**

We collected 29 studies, including 53 memory tasks. The results showed that musicians performed better than nonmusicians in terms of long-term memory, *g* = .29, 95% CI (.08–.51), short-term memory, *g* = .57, 95% CI (.41–.73), and working memory, *g* = .56, 95% CI (.33–.80). To further explore the data, we included a moderator (the type of stimulus presented, i.e., tonal, verbal or visuospatial), which was found to influence the effect size for short-term and working memory, but not for long-term memory. In terms of short-term and working memory, the musicians’ advantage was large with tonal stimuli, moderate with verbal stimuli, and small or null with visuospatial stimuli.

**Conclusions:**

The three meta-analyses revealed a small effect size for long-term memory, and a medium effect size for short-term and working memory, suggesting that musicians perform better than nonmusicians in memory tasks. Moreover, the effect of the moderator suggested that, the type of stimuli influences this advantage.

## Introduction

Musicians are a class of experts amply investigated in recent years to shed light on the effect of musical expertise not only on auditory, but also on cognitive performance across the lifespan [1]. Musicians are more adept than nonmusicians in performing musical tasks, of course, but their performance is often better in classic auditory tasks too, and even in cognitive tasks. For example, musicians perform better than nonmusicians in such auditory tasks as frequency and temporal discrimination [2, 3, 4], in perceiving the prosody of speech [5, 6], or in understanding speech in noisy environments [7]. Interestingly, the literature shows that the superiority of musicians also extends to cognitive skills, such as visuospatial cognition [8, 9], mathematical abilities [10, 11], language [12, 13], and memory in particular (e.g., [14, 15]).

Several studies found that musicians had better memory than nonmusicians, but this was not observed consistently in all memory tasks. Here, the published findings are described separately for long-term, short-term, and working memory, based on the classic distinction between the memory systems (e.g., [16]), (see the Method section for a description of the memory systems and the tasks used to tap them).

In long-term memory tasks, musicians of all ages generally performed better than nonmusicians in verbal learning and recall tasks (involving words and numbers) [14, 17, 18, 19, 20, 21, 22], although a few studies did not find this was true of adult musicians [23, 24, 25]. When the stimuli were visual (e.g., figures), only one study found that adult musicians performed better than nonmusicians [19]. In the remaining studies, there was no difference between musicians’ and nonmusicians’ performance, neither in adults nor in children [17, 20, 21, 23, 24, 26]. Finally, when musical stimuli were used, such as familiar and unfamiliar pop songs, musicians performed better than nonmusicians [20], although no difference emerged between musicians and nonmusicians in one study involving a melody learning and recognition task [27].

In short-term memory tasks, musicians of all ages performed better than nonmusicians when asked to reproduce sequences of numbers, letters, or words [15, 25, 28, 29, 30, 31, 32, 33, 34, 35] but two studies testing adult musicians and nonmusicians did not observe any difference [36, 37]. Adult and older musicians were also found better than nonmusicians at reproducing visual and spatial sequences in some studies [25, 29, 38, 39, 40], but not in others (e.g., [15, 34, 37, 41, 42]). Finally, when the stimuli were musical (e.g., tones, chords, melodies), adult musicians unsurprisingly performed better than nonmusicians [40, 43, 44, 45, 46, 47].

Several researchers also examined how musicians and nonmusicians performed in working memory tasks. Musicians were more successful than nonmusicians in tasks that involved storing and manipulating verbal information or recalling information while completing a secondary task [7, 14, 25 29, 30, 31, 32, 35, 48, 49, 50, 51, 52, 53]. Here again, though, some studies found no such difference, or it emerged for children but not for adults [15, 30, 36, 49, 54]. When the stimuli were visual and spatial, some studies found that musicians fared better than nonmusicians, but only as far as children were concerned [30, 35, 48], whereas this difference was not apparent in adult or elderly study participants [15, 30, 42, 50, 52]. Finally, when musical stimuli were used to test their working memory, adult musicians naturally performed better than nonmusicians [44, 46, 53].

To sum up, differences between musicians and nonmusicians in memory tasks seem to vary as a function of the memory system, the type of stimulus (e.g., verbal, visual, spatial, or tonal), and the age of participants in the various studies. The overall results seem consistent for short-term memory, with numerous studies finding that musicians scored higher than nonmusicians regardless of the type of stimuli involved. But for long-term memory and working memory, the findings across studies paint a less clear picture: musicians outperformed nonmusicians when the stimuli were verbal or tonal, and when the participants were children; results varied when the stimuli were visual and spatial, and the participants were adults. Although some evidence has been collected to indicate that musicians have better memory than nonmusicians (particularly in short-term memory tasks) there are the picture is still not very clear.

The present meta-analysis explored the published studies conducted on young adult musicians and nonmusicians with a view to ascertaining: whether there really is a difference between the two; and whether the magnitude of any difference varies as a function of the memory system involved, and the type of stimuli presented. As concerns the participants’ age, although the literature includes studies on children and older adults (age > 65 years) too, we focused here on young adults because: (i) the studies on young adults are more numerous (N = 29) than those involving children (N = 7) and older people (N = 2); (ii) children will rarely have had many years of music training; and (iii) older adults’ performance in cognitive tasks varies considerably by comparison with that of young adults, often making it more difficult to interpret the findings (e.g., [55]). As for the stimuli, the type of stimulus was considered here as a moderator, which might shed light on whether the memory advantage observed in musicians is domain-specific (i.e., only for tonal stimuli), or generalized to other domains and therefore detectable with verbal and/or visuospatial stimuli too. Since expertise is known to improve domain-specific abilities (see for example [56]), we expected to observe different results depending on the type of stimuli. In particular, we expected to find the largest difference between musicians and nonmusicians when the memory tasks used to test them included tonal stimuli in the musical domain.

## Method

### Study selection

We searched for studies using the AIRE portal, a service provided by Padua University that allows for searches across multiple databases: Education Source; PEP (WEB)—Psychoanalytic Electronic Publishing; Psychology and Behavioral Science (EBSCO); PsycINFO (Ovid); PubMed; ScienceDirect—AllBooks Content (Elsevier API); SCOPUS (Elsevier API); SocINDEX with Full Text (EBSCO); Web of Science. We also used Google Scholar in a subsequent step. The search terms used were: “memory”, “musicians”, “nonmusicians”. Finally, some papers were found by checking the references of previously-identified papers. We also discussed whether or not to include relevant studies in the so-called “grey literature”, but opted to consider only published studies (or those in press). We are aware that this might expose our findings to a “publication bias” (due to the tendency to publish studies producing statistically significant results, not those with a null result) that, in our case, would exaggerate any differences between musicians and nonmusicians [57]). On the other hand, published results have survived a peer review process (and unpublished studies may have not). Any search in the grey literature is also bound to be inconclusive, as it is impossible to be sure of identifying all the relevant unpublished literature available. The latest search for studies for the present analysis was conducted on 15 February 2017.

### Inclusion criteria

Studies were included only if they met all the following criteria: (1) studies that had adults as participants; (2) studies that included a group of expert musicians (i.e., participants who had attended music conservatories or music schools), and a group of nonmusicians (i.e., participants who had little or no experience of playing a musical instrument); (3) studies that administered a memory task to both groups, that could load participants’ long-term, short-term, or working memory (see “Categorization of memory tasks” below for details); (4) studies using stimuli that could be classified as verbal, visual, spatial, or tonal; (5) studies published in English. After excluding the studies that did not match all the above inclusion criteria, further exclusions were made because data were missing (not provided by the authors), or because the tasks administered were not comparable with those of the other studies considered. The PRISMA flow diagram [58] represents all the steps of the literature search (see S1 Fig). Two independent raters coded and assessed the quality of the single studies, and particular whether the characteristics of the participants and tasks were adequately described. Any disagreements between the raters were solved by consulting and discussing the original article. Their assessment was used to screen the studies binarily (pass or fail) for inclusion in the analysis.

### Categorization of memory tasks

We divided the studies on the grounds of the memory system being tapped in the experiments, distinguishing between long-term, short-term, and working memory. Long-term memory stores information for a time ranging from a few minutes to (potentially) a whole life-time; it is usually investigated with recall and recognition tasks after a learning phase. Short-term memory enables information (usually from 5 to 9 items) to be retained for a few seconds (up to about half a minute), and is classically assessed using methods such as forward span tasks (e.g., digit span forward, word span forward, Corsi visuospatial span forward). These tasks involve remembering and exactly reproducing a series of digits immediately after they have been presented. Working memory is used to maintain some information temporarily while manipulating this information (e.g., the backward digit span), or performing a secondary task. An example of the latter case is the operation span task [59], which involves solving increasingly long sets of simple arithmetical operations, each of which is followed by a word, judging whether the solution of the operation is correct or not, and then recalling all the words that followed each operation in the right order. The terms working memory and short-term memory are often used to mean the same concept, but in the present meta-analysis these two systems were kept separate: short-term memory only enables information to be retained, whereas working memory is an active ability to temporarily store and simultaneously process information (e.g., [60, 61]).

We classified the memory tasks as follows. Long-term memory tasks involved the delayed recall or recognition of information. Short-term memory tasks included forward span tasks for both verbal stimuli (words and numbers), and visual and spatial stimuli (figures and spatial positions), and recognition tasks in the case of musical stimuli (tones, melodies, chords). Working memory tasks involved either performing a secondary task as well as the primary recall task, or manipulating the information to be remembered (e.g., backward span tasks). Table 1 shows the complete list of tasks distinguished according to the three memory systems they load.

**Table 1:** List of tasks and the memory systems they tap.

TASK	MEMORY SYSTEM
Berliner Intelligenzstruktur Test—Recognition of two-digit numbers	LONG-TERM MEMORY
Berliner Intelligenzstruktur Test—Recognition of buildings on a city map	LONG-TERM MEMORY
Berliner Intelligenzstruktur Test—Recognition of previously memorized nouns	LONG-TERM MEMORY
Learning and recall of word lists	LONG-TERM MEMORY
Benton Visual Retention test	LONG-TERM MEMORY
Rey–Osterrieth Complex Figure Test—delayed recall	LONG-TERM MEMORY
The Rey Auditory Verbal Learning Test—delayed recall	LONG-TERM MEMORY
Recognition of previously memorized words	LONG-TERM MEMORY
California Verbal Learning Test	LONG-TERM MEMORY
Recognition of previously memorized melodies	LONG-TERM MEMORY
Rey Visual Design Learning Test	LONG-TERM MEMORY
Figure recognition	LONG-TERM MEMORY
Non-words recognition	LONG-TERM MEMORY
Digit span forward	SHORT-TERM MEMORY
Test of Memory and Learning—Digits forward	SHORT-TERM MEMORY
Test of Memory and Learning—Letters forward	SHORT-TERM MEMORY
Test of Memory and Learning—Abstract visual memory	SHORT-TERM MEMORY
Test of Memory and Learning—Memory for location	SHORT-TERM MEMORY
Spatial span forward	SHORT-TERM MEMORY
Non-word span	SHORT-TERM MEMORY
One-back task	SHORT-TERM MEMORY
Non-word repetition	SHORT-TERM MEMORY
Tonal sequence forward	SHORT-TERM MEMORY
Atonal sequence forward	SHORT-TERM MEMORY
Presentation of a sequence of 5 tones—recognition of one tone (tonal)	SHORT-TERM MEMORY
Presentation of a sequence of 5 tones—recognition of one tone (atonal)	SHORT-TERM MEMORY
Static matrix span	SHORT-TERM MEMORY
Syllable span	SHORT-TERM MEMORY
Recognition of consonants	SHORT-TERM MEMORY
Recognition of digits	SHORT-TERM MEMORY
Digit span backward	WORKING MEMORY
Reading span	WORKING MEMORY
Operation span	WORKING MEMORY
Test of Memory and Learning—Digits backward	WORKING MEMORY
Test of Memory and Learning—Letters backward	WORKING MEMORY
Spatial span backward	WORKING MEMORY
Two-back task	WORKING MEMORY
Presentation of a syllable and a sine wave tone simultaneously—tone recognition	WORKING MEMORY
Presentation of a syllable and a sine wave tone simultaneously—syllable recognition	WORKING MEMORY
Digit span forward with articulatory suppression	WORKING MEMORY
Visuospatial span	WORKING MEMORY

Some tasks were used in more than one study.

### Procedure

The preliminary dataset included 37 studies and 99 tasks. For each task, we recorded the variance and Hedges’ *g*, a measure of the effect size adjusted for small groups [62]. The *g* values were interpreted according to the criteria suggested by Cohen (1988): small effect = 0.2 to 0.5; medium effect = 0.5 to 0.8; large effect > 0.8 [63]. The effect size was calculated using raw mean scores, standard deviations, and sample sizes of the group of musicians and the group of nonmusicians. When raw mean scores, standard deviations, and sample sizes were unavailable, the effect size was calculated starting from the value of *F* (Fisher) or *t* (Student’s t-distribution). If none of the above information was provided in the study, or other data were missing (e.g., the number of participants), the authors were contacted. We contacted eleven authors in all, and three provided us with the missing data; the other eight studies were excluded from the meta-analysis.

The final dataset for our meta-analysis thus included 29 studies and 75 tasks. Multiple measures (i.e., tasks) of the same construct (e.g., two different tasks tapping verbal working memory) were used in 15 studies, so the effect sizes of these multiple measures were combined—using the Borenstein method [64], with the *Mad* package [65] of the *R* software [66]—to avoid overestimating these effect sizes in the meta-analysis. This method combines different effect sizes for dependent groups and takes into account the correlation that might exist between two or more non-independent measures. The final dataset thus included 53 tasks (used in the 29 studies) that were divided as follows: 14 tasks (10 studies) assessing long-term memory, 20 tasks (16 studies) assessing short-term memory, and 19 tasks (16 studies) assessing working memory.

We ran a separate meta-analysis for each memory system, with the *R* software and the *Metafor* package [67]. Since the literature suggests that differences between musicians and nonmusicians might be due to the type of stimuli presented, we included this variable as a moderator in the meta-analyses. Stimuli were classified as: verbal (i.e., words, letters, and numbers, either read or heard); visuospatial (i.e., figures; spatial positions of figures); and tonal (i.e., musical tones; melodies). Although some of the literature suggests a distinction between visual and spatial memory (e.g., [68], for working memory), visual and spatial tasks were combined for our purposes because of the limited number of studies including each of these types of task.

For each memory system, we first ran a random-effects model meta-analysis using the restricted maximum likelihood method [69]. We also estimated the summarized Hedges’ g values for each meta-analysis using a Bayesian approach (with the *bayesmeta* package; [70]). As this is the first meta-analysis to compare the memory of musicians and nonmusicians, we selected less informative priors for our model parameters. In particular, for *μ* we used a normal prior with a mean of 0 and a standard deviation of 10, while for *τ* we adopted a uniform prior of parameters 0 and 3. Both the maximum likelihood approach and the Bayesian approach led to the same conclusion (see S1 Table). Next, we explored the heterogeneity across studies using forest plots to obtain a graphical representation. We examined this heterogeneity using the *Q* statistic [71], which is distributed like the chi-square under the null hypothesis, with a significant chi value indicating the presence of heterogeneity across studies. We then estimated the magnitude of the heterogeneity with the *I*^*2*^ index (i.e., the proportion of observed variance that reflects differences in effect sizes, [62]). A high *I*^*2*^ value (i.e., *I*^*2*^ > 75%; [72]) might reveal different results across studies, which can have several reasons: for example, the studies could have measured different constructs or had a different design. In contrast, a low *I*^*2*^ (i.e., *I*^*2*^ < 50%; [72]) value might reflect similar results across studies, which can therefore represent a true, generalizable effect.

We also considered the presence of publication bias in each of the three meta-analyses (i.e., long-term, short-term, and working memory). Publication bias [73] is the phenomenon that makes studies reporting a statistically significant result (e.g., a difference between groups) more likely to be published than studies reporting a null result (e.g., no difference between groups). We assessed the publication bias using the funnel plot with the “trim and fill” method [62, 74].

To investigate the robustness of our results we ran a sensitivity analysis using the “leave-one-out” method [69], which computes several meta-analyses, leaving one study out each time. If the mean effect size changes substantially when a given study is removed, this means that the value of the mean effect size does not reflect the true mean, and that the studies lack homogeneity.

Finally, the role of the type of stimulus as a moderator was examined using mixed-effects models (i.e., the type of stimulus was included as a fixed effect). The effect of the moderator was tested using Wald’s chi-square [69]. Pairwise planned comparisons were used as well to explore the difference between the levels of the moderator. These comparisons were not orthogonal, so the type I error was controlled using the false discovery rate [75]. Table 2 shows the estimated means and 95% confidence intervals of the mixed-effects model.

**Table 2:** Analysis of the moderating effect of the type of stimuli by memory system.

Memory system	Tonal(*95% CI*)	Verbal(*95% CI*)	Visuospatial(*95% CI*)	Pairwise comparisons
Long-Term Memory	.01	**.44**	.12	No difference
	(-1.03–1.04)	**(.16–.73)**	(-.22–.45)	
	n = 1	n = 8	n = 5	
Short-Term Memory	**1.15**	**.54**	**.28**	Ton > Verb
	**(.79–1.51)**	**(.38–.71)**	**(.04–.52)**	Ton > Vis
	n = 4	n = 11	n = 5	
Working Memory	**1.04**	**.59**	.01	Ton > Vis
	**(.48–1.60)**	**(.34–.84)**	(-.50–.52)	
	n = 3	n = 13	n = 3	

Estimated mean, 95% confidence intervals (*CI*) of summarized Hedges’ *g*, and number of tasks by memory system and type of stimuli, calculated with the mixed-effects random models. Effect sizes significantly different from 0 at *p* < .05 are shown in bold. Significant pairwise differences between levels of the type of stimuli are displayed in the last column (i.e., pairwise comparisons). Ton = tonal; Verb = verbal; Vis = visuospatial.

## Results

### Descriptive statistics

The studies included in our meta-analysis were conducted between 1987 and 2017. The mean age of participants was 23.38 years (*SD* = 4.67). The samples varied in size between 20 and 140 participants (*mean* = 45.96, *SD* = 23.39), and were always divided into two groups: musicians and nonmusicians. Studies reported the duration of the musicians’ music training in different ways: some reported the minimum years of music training, others the mean years of music training, and some reported both. Across the studies that provided this information, the minimum duration of music training was four years, while the average was 13.73 years. Table 3 shows the effect sizes of each task included in the three meta-analyses.

**Table 3:** Effect sizes and details of each task included in the three meta-analyses.

AUTHORS	YEAR OF PUBLICATION	MEMORY SYSTEM	TYPE OF STIMULI	*n* M	*n* NM	*g*	Var	Mean age (*yrs*)
Anaya, Pisoni & Kronenberger	2016	STM	VERBAL	24	24	.52	.086	22.08
Bialystock & De Pape	2009	STM	VISUOSPATIAL	22	24	.42	.086	24.25
Bialystock & De Pape	2009	WM	VISUOSPATIAL	22	24	.39	.086	24.25
Boebinger & Evans	2015	STM	VERBAL	25	25	.19	.080	27.2
Boebinger & Evans	2015	WM	VERBAL	25	25	.30	.081	27.2
Brandler & Rammsayer	2003	LTM	VERBAL	35	35	.19	.044	28.45
Brandler & Rammsayer	2003	LTM	VISUOSPATIAL	35	35	-.06	.057	28.45
Chan, Ho, & Cheung	1998	LTM	VERBAL	30	30	.93	.056	19.75
Chan, Ho, & Cheung	1998	LTM	VISUOSPATIAL	30	30	.18	.050	19.75
Clayton et al.	2016	WM	VERBAL	17	17	1.01	.127	23.5
Franklin et al.	2008	LTM	VERBAL	12	13	.57	.119	19.73
Franklin et al.	2008	WM	VERBAL	11	9	.95	.170	21.6
George & Coch	2011	STM	VERBAL	16	16	.62	.098	20.25
George & Coch	2011	WM	VERBAL	16	16	.60	.098	20.25
George & Coch	2011	STM	VISUOSPATIAL	16	16	.56	.098	20.25
Hansen, Wallentin, & Vuust	2012	STM	VERBAL	20	20	.97	.112	21.05
Hansen, Wallentin, & Vuust	2012	WM	VERBAL	20	20	-.06	.100	21.05
Hansen, Wallentin, & Vuust	2012	STM	VISUOSPATIAL	20	20	.42	.102	21.05
Hansen, Wallentin, & Vuust	2012	WM	VISUOSPATIAL	20	20	-.21	.101	21.05
Helmbold, Rammsayer & Altenmueller	2005	LTM	VERBAL	70	70	.06	.021	22.5
Helmbold, Rammsayer & Altenmueller	2005	LTM	VISUOSPATIAL	70	70	-.03	.029	22.5
Huang et al.	2010	LTM	VERBAL	10	10	.90	.220	21.45
Jakobson, Lewycky, Kilgour, & Stoesz	2008	LTM	VERBAL	15	21	.87	.083	19
Jakobson, Lewycky, Kilgour, & Stoesz	2008	LTM	VISUOSPATIAL	15	21	.82	.093	19
Lee, Lu, & Ko	2007	STM	VERBAL	20	20	.58	.078	22
Lee, Lu, & Ko	2007	WM	VERBAL	20	20	-.31	.077	22
Lee, Lu, & Ko	2007	WM	VISUOSPATIAL	20	20	-.17	.100	22
Monahan, Kendall, & Carterette	1987	STM	TONAL	12	10	1.02	.193	n.d.
Okhrey, Kutsenko, & Makarchuk	2017	STM	VERBAL	28	36	.29	.046	20
Okhrey, Kutsenko, & Makarchuk	2017	STM	VISUOSPATIAL	28	36	-.27	.062	20
Pallesen et al.	2010	STM	TONAL	11	10	1.42	.239	26.5
Pallesen et al.	2010	WM	TONAL	11	10	.51	.197	26.5
Parbery-Clark, Strait, Anderson, & Hittner	2011	WM	VERBAL	18	19	1.30	.126	50
Ramachandra, Meighan, & Gradzki	2012	STM	VERBAL	30	30	.78	.054	19.45
Ramachandra, Meighan, & Gradzki	2012	WM	VERBAL	30	30	.72	.053	19.45
Rodrigues, Loureiro, & Caramelli	2014	STM	VISUOSPATIAL	38	38	-.14	.040	32.15
Schiavo & Timmers	2016	LTM	TONAL	10	10	.01	.183	24.75
Schulze et al.	2011	WM	TONAL	16	17	1.44	.153	24.49
Schulze et al.	2011	WM	VERBAL	16	17	.43	.124	24.49
Schulze, Dowling, & Tillman	2012	STM	TONAL	20	20	.96	.084	22.68
Schulze, Dowling, & Tillman	2012	WM	TONAL	20	20	1.08	.086	22.49
Schulze, Mueller, & Koelsch	2011	STM	TONAL	16	17	1.37	.114	24.49
Suàrez, Elangovan, & Au	2016	WM	VERBAL	24	30	.62	.079	22.59
Suàrez, Elangovan, & Au	2016	STM	VISUOSPATIAL	24	30	.45	.058	22.59
Suàrez, Elangovan, & Au	2016	STM	VERBAL	24	30	.43	.077	22.59
Suàrez, Elangovan, & Au	2016	LTM	VERBAL	24	30	-.19	.075	22.59
Talamini, Carretti & Grassi	2016	STM	VERBAL	18	18	.66	.079	22.6
Talamini, Carretti & Grassi	2016	WM	VERBAL	18	18	.36	.075	22.6
Taylor & Dewhurst	2017	LTM	VERBAL	20	20	.66	.101	21.67
Vasuki, Sharma, Demuth, & Arciuli	2016	STM	VERBAL	17	18	.58	.114	25.75
Vasuki, Sharma, Demuth, & Arciuli	2016	WM	VERBAL	17	18	.14	.128	25.75
Weiss, Biron, Lieder, Granot, & Ahissar	2014	STM	VERBAL	42	15	.54	.093	23.35
Zuk, Benjamin, Kenyon, & Gaab.	2014	WM	VERBAL	15	15	1.19	.157	24.8

The effect size is expressed as Hedges’ *g*. For each task, additional information is provided on the authors, the year of publication of the study, the memory system investigated, the type of stimuli presented in the memory task, the number of participants, and the mean age of participants. LTM = long-term memory; STM = short-term memory; WM = working memory; M = musicians; NM = nonmusicians.

### Long-term memory

**Fig 1:**
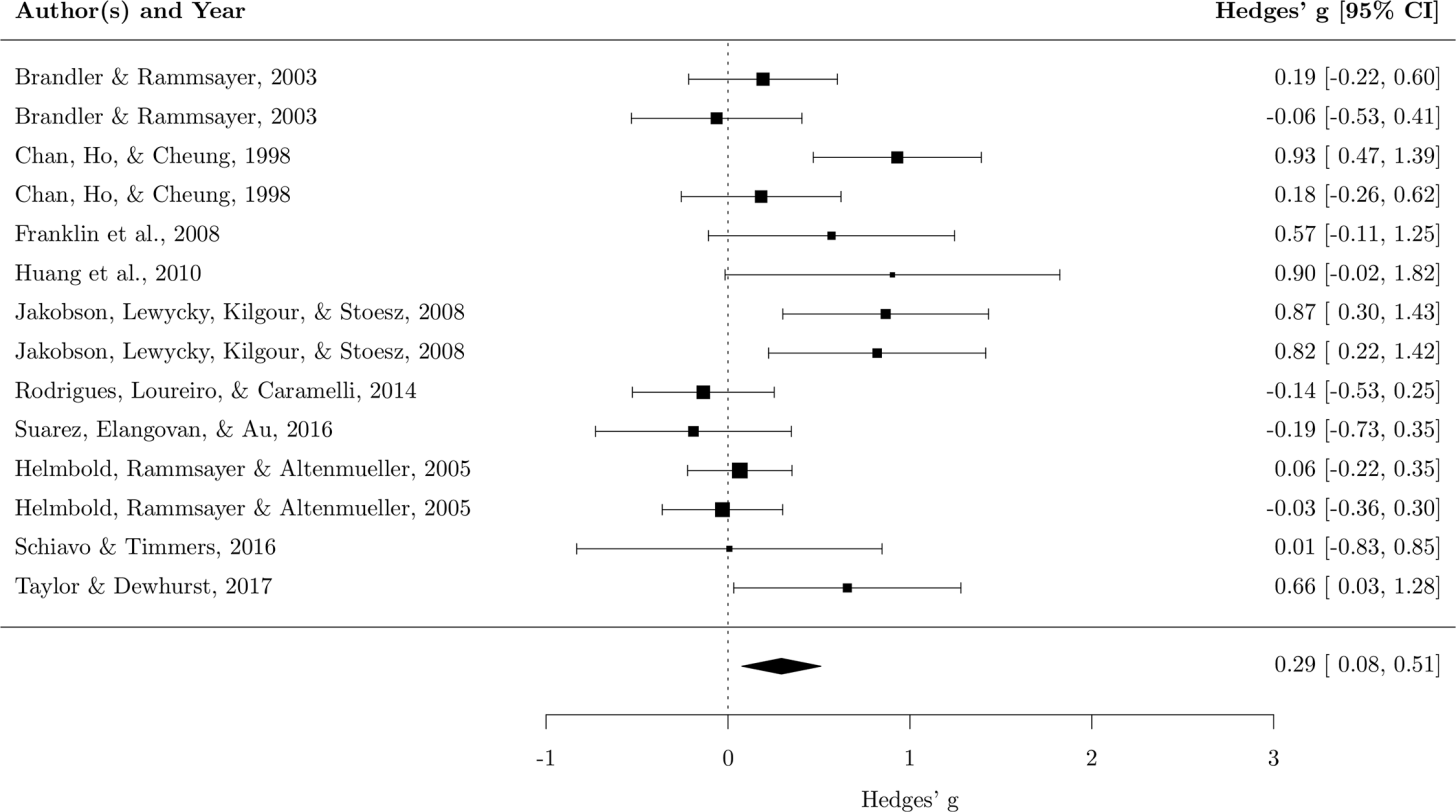
Forest plot for long-term memory. Each square represents the effect size of the study together with 95% confidence interval. The size of the symbol is proportional to the study’s weight.

**Fig 2:**
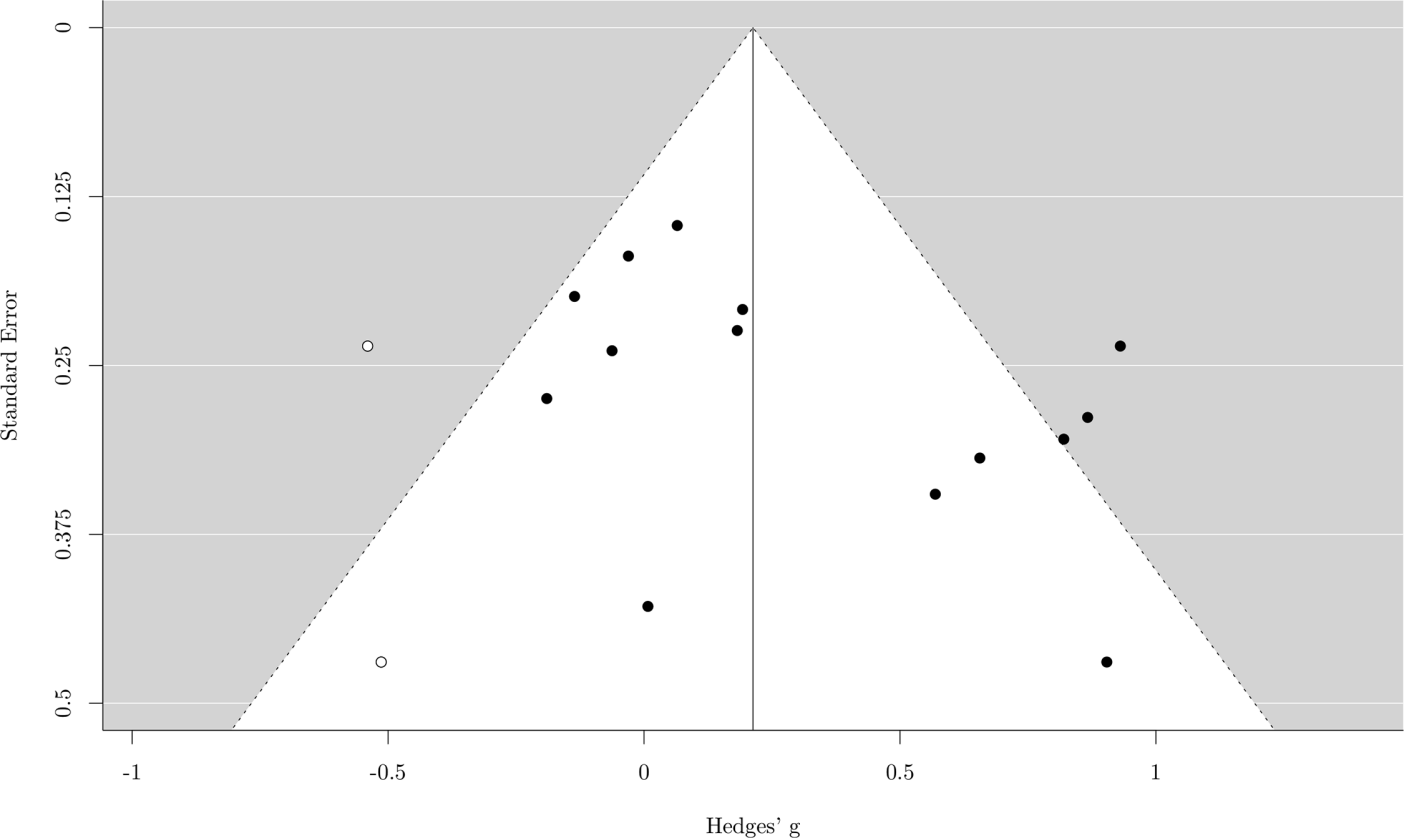
Funnel plot for long-term memory. Each black dot represents one study included in the meta-analysis. Any white dots represent the effect size of hypothetical unpublished results.

The random effect analysis showed a small mean effect size, *g* = .29, 95% *CI* (.08–.51), *p* = .008, meaning that musicians tended to perform better than nonmusicians in long-term memory tasks. The heterogeneity was significant, *χ*^*2*^ (13) = 33.45, *p* = .001, *I*^*2*^ = 63.71%, suggesting that the results of different studies exhibited a moderate variance (Fig 1). The sensitivity analysis showed that the mean effect size did not vary consistently. In fact, the *g* value varied between .22 and .33 (*mean* = .29, *SD* = .03), so the effect size remained small regardless of which study was excluded. We also assessed the publication bias, and the funnel plot with trim and fill added two hypothetical missing studies (Fig 2). Including these two studies reduced the effect size, which was no longer significant, *g* = .21, 95% *CI (*-.02–.44), *p* = .068.

The test of the moderator was not significant: *χ*^*2*^ (2) = 2.42, *p* = .298 (for more details see, Table 2).

### Short-term memory

**Fig 3:**
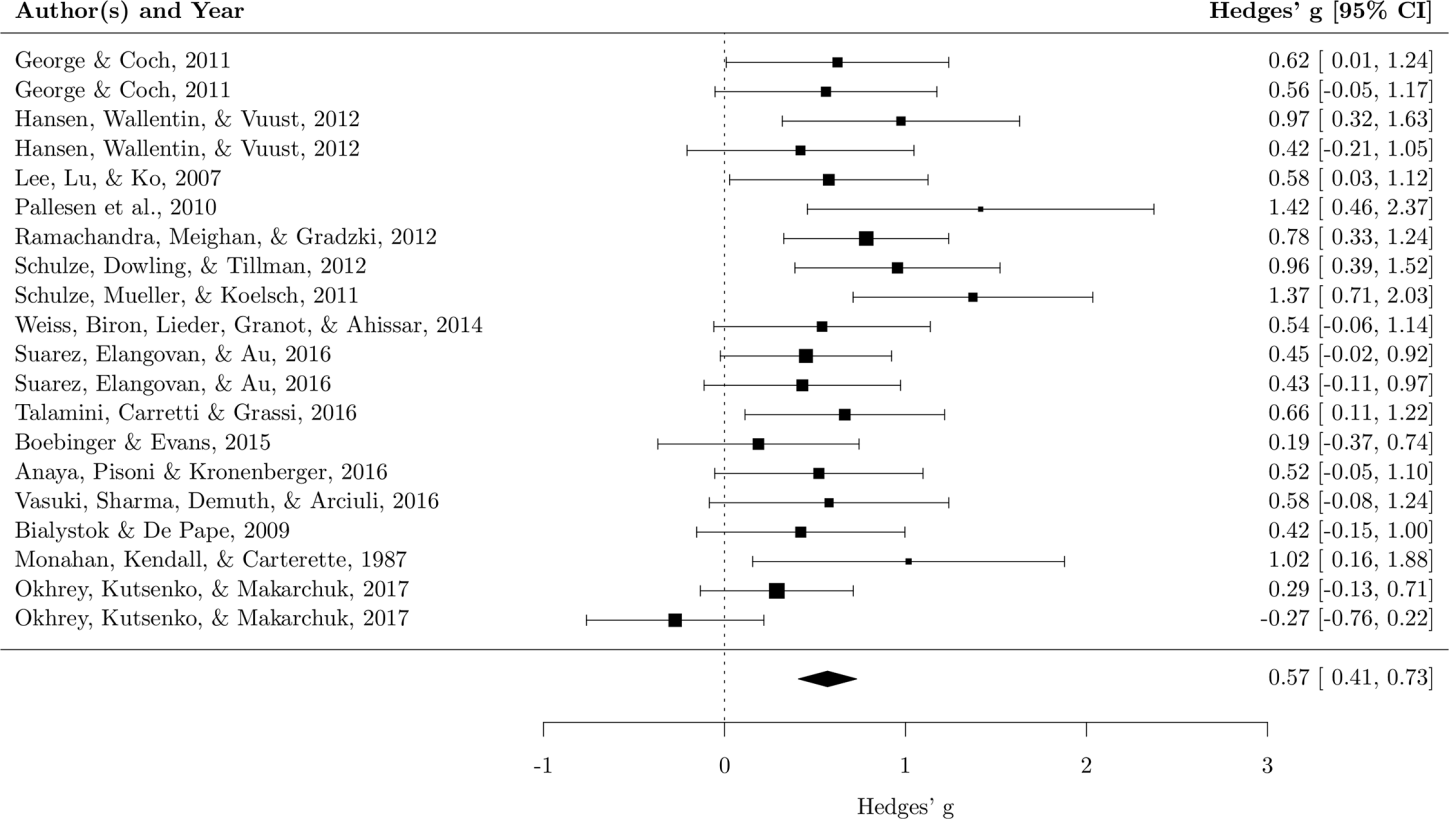
Forest plot for short-term memory. Each square represents the effect size of the study together with the 95% confidence interval. The size of the symbol is proportional to the study’s weight.

**Fig 4:**
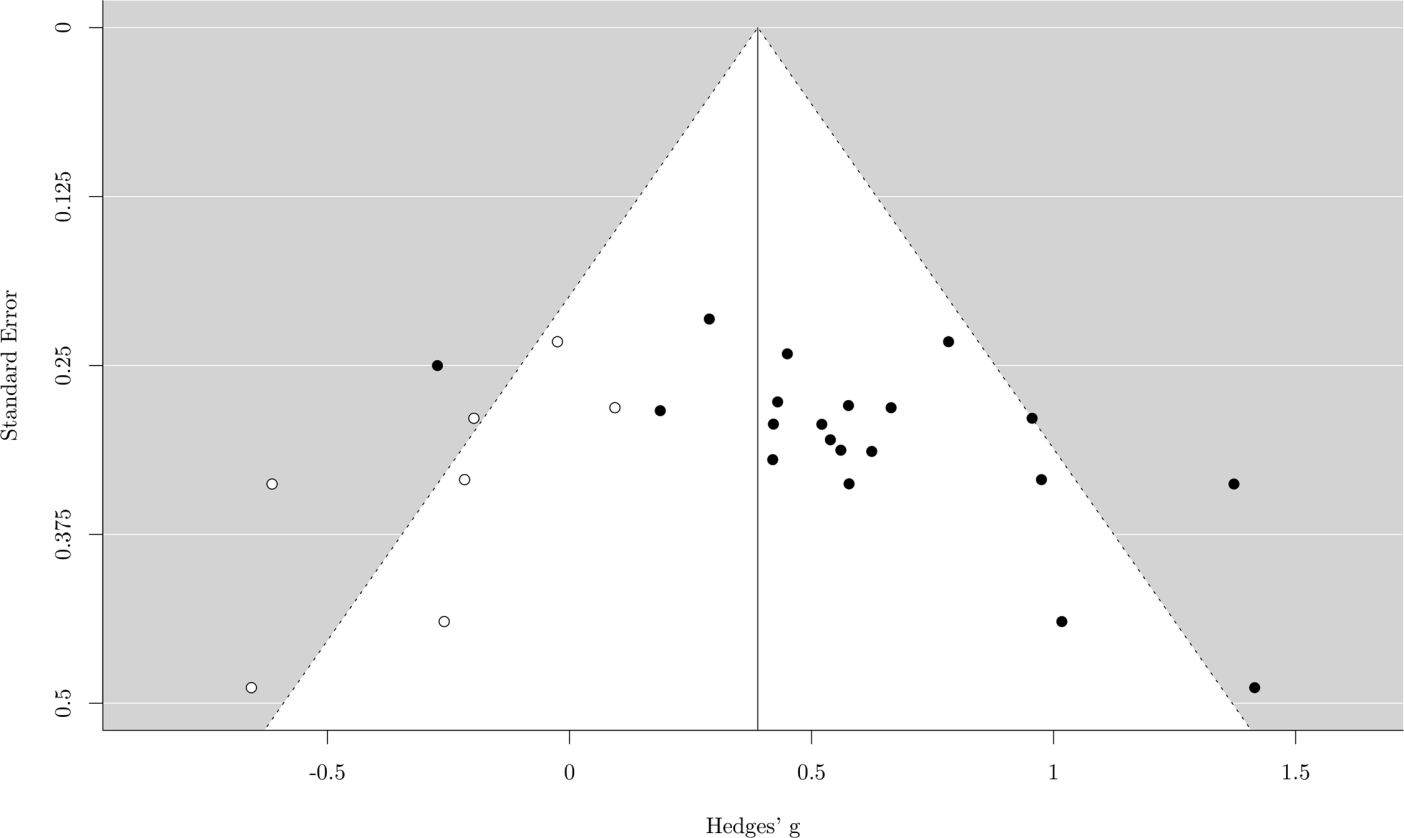
Funnel plot for short-term memory. Each black dot represents one study included in the meta-analysis. Any white dots represent the effect size of hypothetical unpublished results.

The random effect analysis showed a moderate mean effect size, *g* = .57, 95% *CI* (.41–.73), *p* < .001, meaning that musicians performed better than nonmusicians in short-term memory tasks. The heterogeneity was not significant, *χ*^*2*^ (19) = 29.67, *p* = .056, *I*^*2*^ = 35.36%, suggesting that most of the studies produced similar results (Fig 3). The sensitivity analysis showed that the mean effect size was robust. In fact, the *g* value ranged between .53 and .61 (*mean* = .57, *SD* = .02), depending on which study was excluded. The funnel plot with trim and fill, used to assess publication bias, added seven hypothetical missing studies (Fig 4), but including these hypothetical studies in the analysis made little difference to the effect size, which remained moderate, *g* = .39, 95% *CI* (.21–.57), *p* < .001.

Although the heterogeneity was not significant (i.e., low variance in the results across studies), we investigated whether the moderator could influence the effect size: the test on the moderator was significant, *χ*^*2*^ (2) = 15.64, *p* < .001, and the heterogeneity was still not significant, *χ*^*2*^ (17) = 14.02, *p* = .66, *I*^*2*^ = 0.04%. The amount of heterogeneity decreased, however, suggesting that the type of stimuli had a role in determining the small differences observed across studies. Specifically, for all the levels of the moderator, the effect size was statistically different from zero, with tonal stimuli showing the largest effect size, and verbal and visuospatial stimuli showing a moderate effect size (see Table 2).

### Working memory

**Fig 5:**
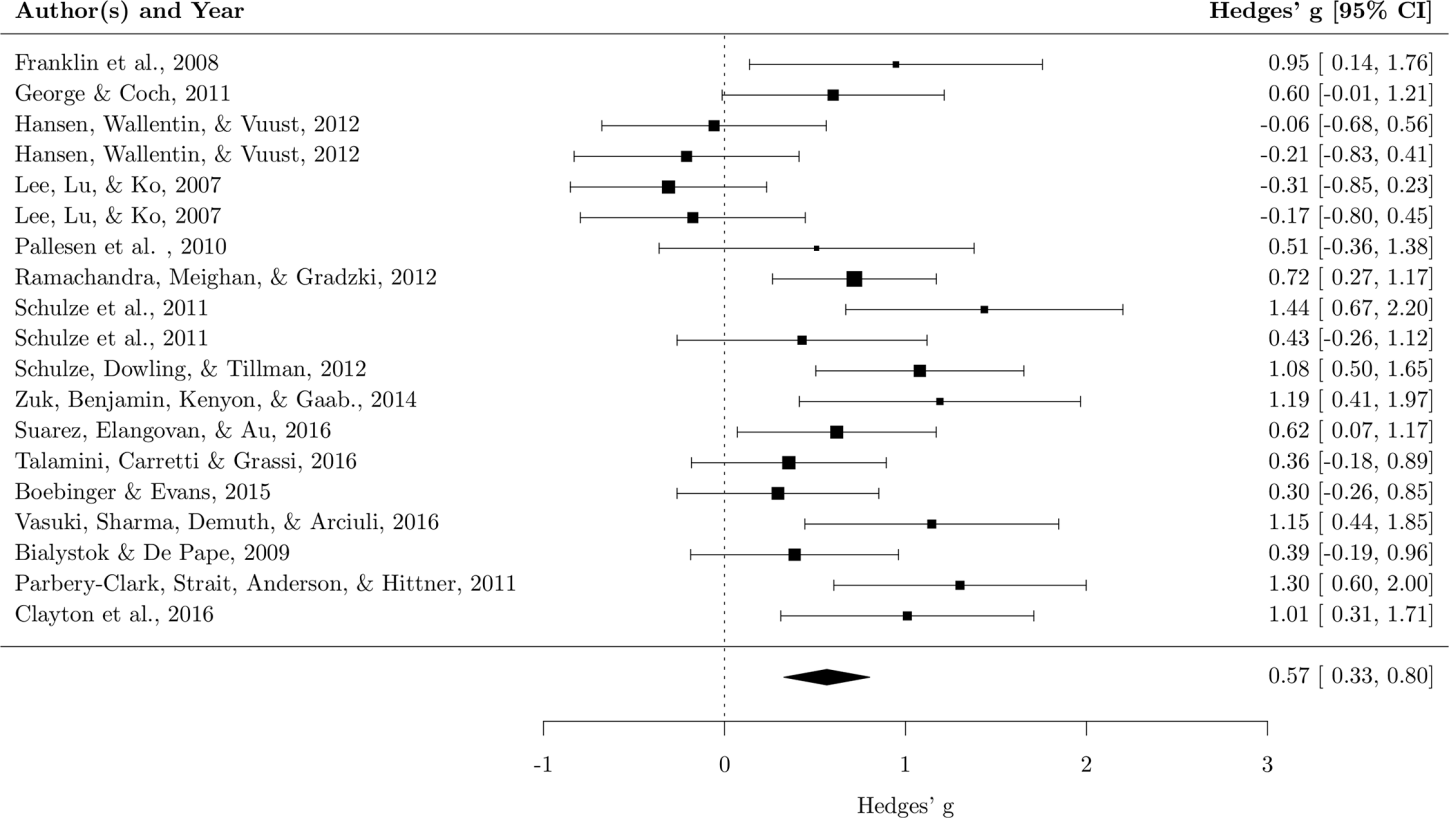
Forest plot for working memory. Each square represents the effect size of the study together with the 95% confidence interval. The size of the symbol is proportional to the study’s weight.

**Fig 6:**
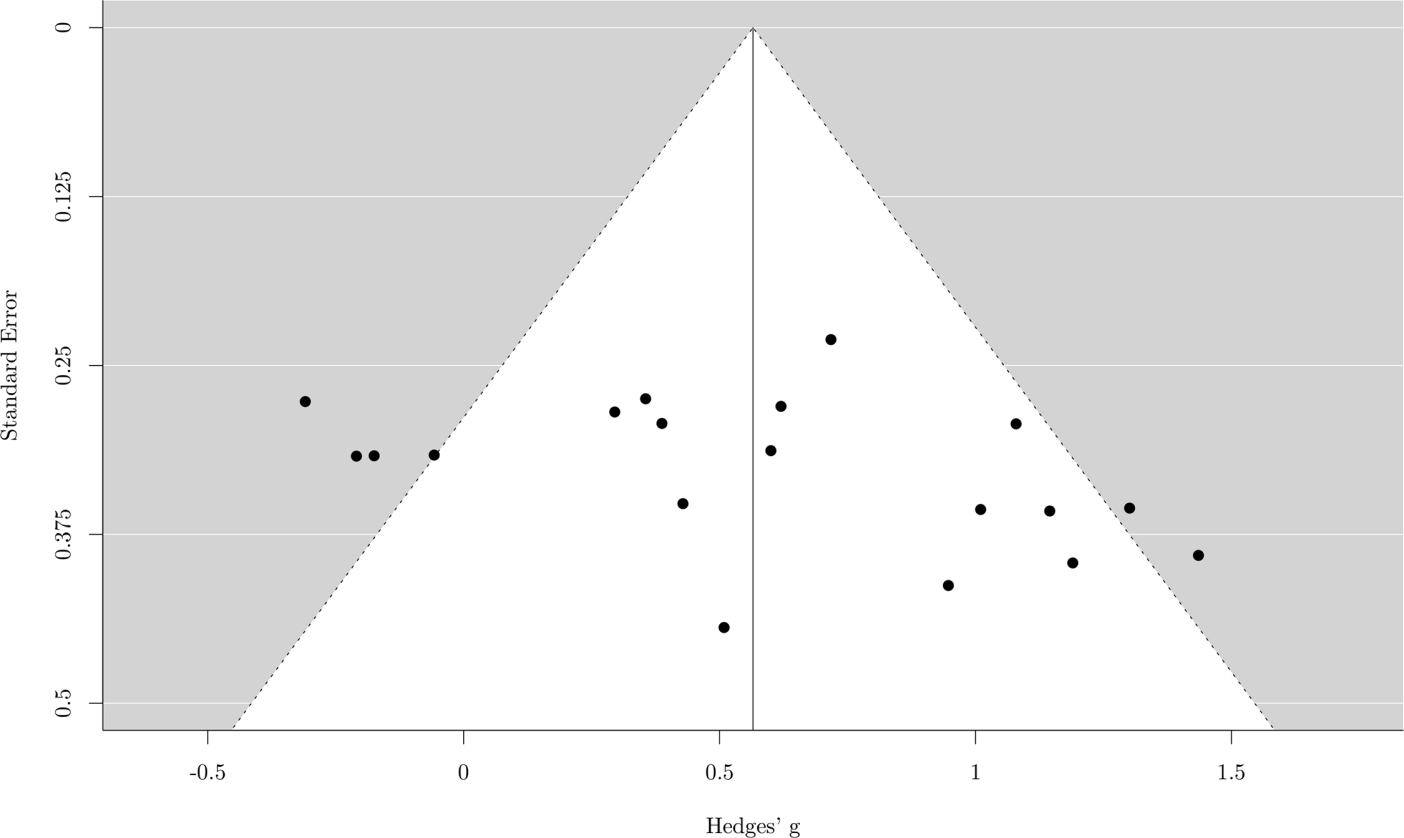
Funnel plot for working memory. Each black dot represents one study included in the meta-analysis. Any white dots represent the effect size of hypothetical unpublished results.

The random effect analysis showed a moderate mean effect size, *g* = .56, 95% *CI* (.33–.80), *p* < .001, meaning that musicians performed better than nonmusicians in working memory tasks. The test of heterogeneity was also significant, *χ*^*2*^ (18) = 47.41, p < .001, *I*^*2*^ = 62.85%, revealing a moderate variance across studies (Fig 5). The sensitivity analysis showed that the mean effect size was robust. In fact, the *g* value varied from .52 to .62 (*mean* = .56, *SD* = .03), showing that none of the studies had a substantial influence on the mean effect size. A funnel plot with trim and fill produced no evidence of publication bias (Fig 6).

The analysis of the moderator showed a significant effect in the case of working memory, *χ*^*2*^ (2) = 7.36, *p* = .025. However, the test for residual heterogeneity was still significant, *χ*^*2*^ (16) = 32.73, *p* = .008, *I*^*2*^ = 51.42%, suggesting that the moderator could explain only a small portion of the variance across studies. Specifically, two of the three levels of the moderator had an associated effect size significantly different from zero: tonal stimuli were associated with the largest effect size, followed by verbal stimuli (moderate effect size). No effect was found (i.e., no difference between musicians and nonmusicians) for visuospatial stimuli (see Table 2 for details).

## Discussion

The present meta-analysis was conducted to investigate whether musicians have better memory than nonmusicians, separately considering the case of long-term memory, short-term memory, and working memory. We also examined whether a possible memory advantage of musicians over nonmusicians could be modulated by the type of stimuli presented in the memory tasks (i.e., verbal, visuospatial, and tonal). As emerged from our literature review in the introduction, musicians often perform better than nonmusicians in various cognitive domains (including memory). According to the literature on musicians’ memory performance, it could be hypothesized that their performance in memory tasks is enhanced for domain-specific (i.e., musical) stimuli, with which they are familiar (e.g. [76]). We conducted our meta-analysis by investigating memory tasks that tap different memory systems using different types of stimuli. Overall, the findings are consistent with a domain-specific superiority of musicians over nonmusicians in memory tasks. The domain specificity hypothesis is not enough to explain all the reported results, however, which are discussed below, by memory system and type of stimuli.

*Long-term memory*. The meta-analysis revealed a slight superiority of musicians over nonmusicians, with a moderate variability across studies. The funnel plot revealed two hypothetical unpublished studies with null or opposite results (i.e., with nonmusicians performing better than musicians), pointing to a possible publication bias. When these two hypothetical studies were included, the difference between musicians and nonmusicians decreased and was no longer significant. Further studies are consequently needed on long-term memory to clarify whether the difference identified between musicians and nonmusicians is genuine. In addition, the moderator was unable to explain the variance across studies, i.e., the heterogeneity, which decreased only slightly after the moderator was introduced in the analysis. It is worth noting that, unlike the case of the studies on short-term and working memory, only one study in the long-term memory meta-analysis investigated the recall of tonal stimuli (i.e., [27]). Participants were asked to learn and remember short ambiguous melodies, and the authors found no difference between musicians and nonmusicians, whereas studies using musical stimuli to test short-term and working memory identified differences with large effect sizes. The shortage of studies using musical stimuli to test long-term memory might be one of the reasons behind the null effect of the moderator in the meta-analysis. The difference between the methodologies used in the studies might also be responsible for the heterogeneity. On the other hand, although the test of moderator was not significant, we found that verbal stimuli were associated with a larger effect size than visuospatial stimuli—a result in line with the picture seen for working memory.

*Short-term memory*. The meta-analysis revealed a moderate effect size, showing that musicians have better short-term memory skills than nonmusicians, with no significant heterogeneity across studies. Here too, the funnel plot suggested a publication bias. By adding the hypothetical missing studies, the effect size remained moderate, strengthening the reliability of the result of the meta-analysis for short-term memory. The moderator analysis revealed a significant effect of the type of stimuli. When the moderator was included, moreover, the already statistically insignificant heterogeneity almost disappeared completely. In other words, the musicians’ advantage changes as a function of the type of stimuli presented in the task: as the domain specificity hypothesis suggests, musicians had an advantage over nonmusicians especially when recalling music stimuli. It is noteworthy, however, that they performed better with verbal and visuospatial stimuli too.

*Working memory*. The moderate effect size resulting from the meta-analysis on working memory shows that musicians have better working memory than nonmusicians. The results across studies revealed a moderate variability, however: some studies reported a superiority of musicians over nonmusicians, while others did not (or not as much). When we included the moderator in the analysis, the heterogeneity was still significant, but decreased slightly, meaning that the type of stimuli presented explained only a small part of the variability across studies. Tonal stimuli were associated with the largest effect size, again supporting a domain-specific advantage. The musicians’ advantage extended to verbal stimuli too (which were associated with a moderate effect size), but not to visuospatial stimuli in this case. We also observed no publication bias.

The present meta-analysis suggests that musicians have better memory than nonmusicians. We might wonder, first of all, whether this difference is genuine or depends on the journals’ policy to publish positive (rather than null) results (e.g., [57]). Paradoxically, if we were to assume that the currently-available literature only contains positive results, then the outcome of our meta-analysis would only reflect a publication bias. By the same token, if this were true, the statistical strategies adopted here to check for publication bias would be of little use. While we cannot exclude that such a bias exists, some of the results analyzed here support the impression that musicians really do have better memory than nonmusicians. For instance, we saw a difference between the effect sizes for long-term (small) and for short-term and working memory (moderate). In addition, the effect of the moderator (the musicians’ advantage was large for tonal stimuli, medium for verbal stimuli, and small-to-null for visuospatial stimuli) reinforces the conviction that the results we observed at least partly reflect a real difference.

That said, the present meta-analysis indicated that musicians perform better than nonmusicians in memory tasks, and this raises the question of why musicians should have better memory than nonmusicians? The memory advantage for tonal stimuli is easily explained in the light of the literature on experts’ performance: experts (musicians in the case in point) perform better than non-experts (i.e., nonmusicians) with stimuli they are familiar with. What remains to be seen is why musicians perform better than nonmusicians in recalling verbal stimuli and (to some extent) visuospatial stimuli too.

We can hypothesize two types of explanation of this situation. On the one hand, there may be some uncontrolled variable, typical of quasi experiments, responsible for the difference in recall performance. For instance, musicians might perform better than nonmusicians because of a sort of Pygmalion (or Rosenthal) effect [77]. If researchers expected musicians to do better, this could induce an improvement in their performance. But this would fail to explain why a difference between musicians and nonmusicians is evident for memory, but not for certain other cognitive abilities (see [78] for a broad overview). Another possibility is that individuals with better memory are more likely to become musicians, and that is why musicians perform better than nonmusicians in memory tasks. The same hypothesis can be applied to any individual characteristic that might help a participant to do well in memory tasks (e.g., enhanced sensory abilities, intelligence, personality, etc.) [79]. Any of these possible explanations would give musicians a constant advantage over nonmusicians across the various memory systems and types of stimuli, but this was not the case in our meta-analysis.

On the other hand, a better memory might be a consequence of having trained to become a musician. Learning to play a musical instrument might improve an individual’s recall of tonal stimuli (according to the domain specificity hypothesis, [80]), and this would explain why musicians outclass nonmusicians in memory tasks involving tonal stimuli. However, our findings suggest that the advantage of musicians extends to verbal stimuli too. As mentioned in the introduction, musicians process auditory stimuli better than nonmusicians [2, 3, 4]. This ability could be helpful in memory tasks, when stimuli are presented orally, because a better auditory encoding of the item to be remembered could strengthen the trace of the stimulus in the listener’s memory. This might explain why musicians perform better than nonmusicians with verbal material too (the stimulus modality hypothesis). In memory tasks, verbal stimuli (e.g., words, numbers, etc.) are often presented orally (as in most of the cases considered in our meta-analysis). This hypothesis finds support, for instance, in a study by Okhrei and colleagues (2017), who found no difference between groups in short-term memory tasks when verbal stimuli were presented visually [37]. Similar results were reported by Talamini and colleagues, (2016), when digits were presented either orally or visually: musicians performed better than nonmusicians in a digit span task when the digits were presented orally, but much less so when they were presented visually [32].

Another possible explanation for the advantage of musicians relating to verbal stimuli concerns the relationship between music and language, as claimed by some authors (see [81, 82] for an overview). Music perception skills are related to phonological awareness and early reading development (e.g. [83, 84, 85]). Music perception skills predict reading skills, even when the variance shared with phonological awareness is removed, suggesting that music perception skills are related to auditory or cognitive mechanisms beyond those tapped by phonological awareness [83]. However, none of the above hypotheses can explain why musicians’ short-term memory fares better with visuospatial stimuli

Finally, the superior performance of musicians might also attributable to the multisensorial nature of music training. Learning to play a musical instrument involves associating the music notation with the sound of the notes, and the motor response. The individual first learns (i.e., by means of specific exercises) to associate music notation with sounds and motor actions. This particular type of training is initially effortful, and demands attentional control. After a while, however, the need for attentional control over the learning process decreases, as the person learns to associate notes, sounds and actions more automatically. Music training might therefore enhance an individual’s active and controlled learning skills, which would be helpful when remembering stimuli in other kinds of memory task too. In other words, music training may nurture active learning strategies, such as chunking. In fact, when learning a sheet of music, chunking is essential in order to commit a melody to memory. Since chunking improves the capacity to memorize series of items, it may be that musicians perform better than nonmusicians in short-term and working memory tasks because they use more efficient chunking strategies. These hypotheses could be investigated by means of appropriate experiments, such as longitudinal studies with participants randomly assigned to different groups (e.g., music training as opposed to other training activities).

We would like to mention some limitations of the present study. The first concerns the number of studies analyzed. We included 29 studies, which were divided into three groups, and submitted to three separate meta-analyses. This meant that we had a limited number of studies for each moderator level (visuospatial, tonal, verbal stimuli), so some levels of the moderator were under-represented (e.g., tonal stimuli in long-term memory). Our results should consequently be interpreted with caution.

The second limitation of the current study is that we could not control for the years of music training because studies reported this information in various different ways. Some studies mentioned the average of the total years of music training; others reported only the minimum number of years of music training. We were consequently unable to include this variable in the meta-analysis, though it could be important in explaining part of the heterogeneity observed across studies. There is currently no standard for describing the characteristics of musicians and nonmusicians, and several potentially interesting characteristics are very often not reported (e.g., hours of daily practice, instrument played, etc.). There are examples in the literature of questionnaires that can be used to draw up a complete profile of participants (both musicians and nonmusicians, [86, 87]) and, since most studies comparing musicians with nonmusicians are quasi experimental, a thorough description of the two groups would be of fundamental importance. In many circumstances, a shortage of information makes it impossible to disentangle whether or not musicians’ enhanced performance is an effect of their music training. Studies also often failed to report or control for variables that might explain the difference between groups: for example, not all the studies analyzed here controlled for general cognitive abilities (e.g., intelligence).

Despite these limitations of the present study, we believe that our work may help to underscore the weaknesses of past studies comparing musicians with nonmusicians so that they might be limits overcome in future research. As already mentioned, longitudinal studies on music training might shed more light on the possible effects of the training per se on a musician’s cognitive abilities. Classic quasi-experimental studies comparing musicians and nonmusicians should provide as many details as possible on their participants and control for general cognitive abilities, socio-economic status, and personality, in order to take into account potential pre-existing differences.

To conclude, this meta-analysis showed that musicians perform better than nonmusicians in memory tasks. Although we have listed several possible explanations for this, none of them seem able to explain all the results. It is likely that more than one mechanism lies behind the musicians’ advantage, and that their better performance is partly domain-specific (for tonal stimuli). It may also be thanks to musicians’ enhanced auditory perception that their recall advantage extends to verbal memory tasks, in which stimuli are often presented orally.

## Supporting information

S1 Fig Details of study selection represented by the PRISMA flow diagram. (DOC)

S1 Table Summary results of meta-analysis by memory system and estimation method. (DOCX)
