# Correction: Musicians have better memory than nonmusicians: A meta-analysis

**DOI:** 10.1371/journal.pone.0191776

**Published:** 2018-01-19

**Authors:** Francesca Talamini, Gianmarco Altoè, Barbara Carretti, Massimo Grassi

The caption for the PRISMA Checklist in the Supporting Information files is incorrect. The caption should read: **S1 File.** PRISMA 2009 Checklist.

S1 Fig does not appear in the Supporting Information files. Please find the S1 Fig below.

## Supporting information

S1 Fig Details of study selection represented by the PRISMA flow diagram. (TIF)
