# Supplementary material for: Musicians have better memory than nonmusicians: A meta-analysis
Source: PLoS One. 2017 Oct 19;12(10):e0186773. doi: 10.1371/journal.pone.0186773 (PMC5648224; doi:10.1371/journal.pone.0186773)
Supplement: S1 Table — (DOCX) [file pone.0186773.s002.docx]

| **Memory System** | **Number of**  **Tasks** | **Maximum Likelihood Approach** | **Bayesian**  **Approach** |
| --- | --- | --- | --- |
|  |  | *Hedges' g*  (95% Confidence Interval) | *Hedges' g*  (95% Bayesian Credible Interval) |
| Long-Term Memory | 14 | .293 (.076 - .511) | .290 (.051 - .548) |
| Short-Term Memory | 20 | .569 (.408 - .730) | .567 (.400 - .744) |
| Working Memory | 19 | .565 (.328 - .802) | .564 (.309 - .827) |

S1 Table
