# Supplementary figures and images for: Correction: Musicians have better memory than nonmusicians: A meta-analysis
Source: PLoS One. 2018 Jan 19;13(1):e0191776. doi: 10.1371/journal.pone.0191776 (PMC5774829; doi:10.1371/journal.pone.0191776)

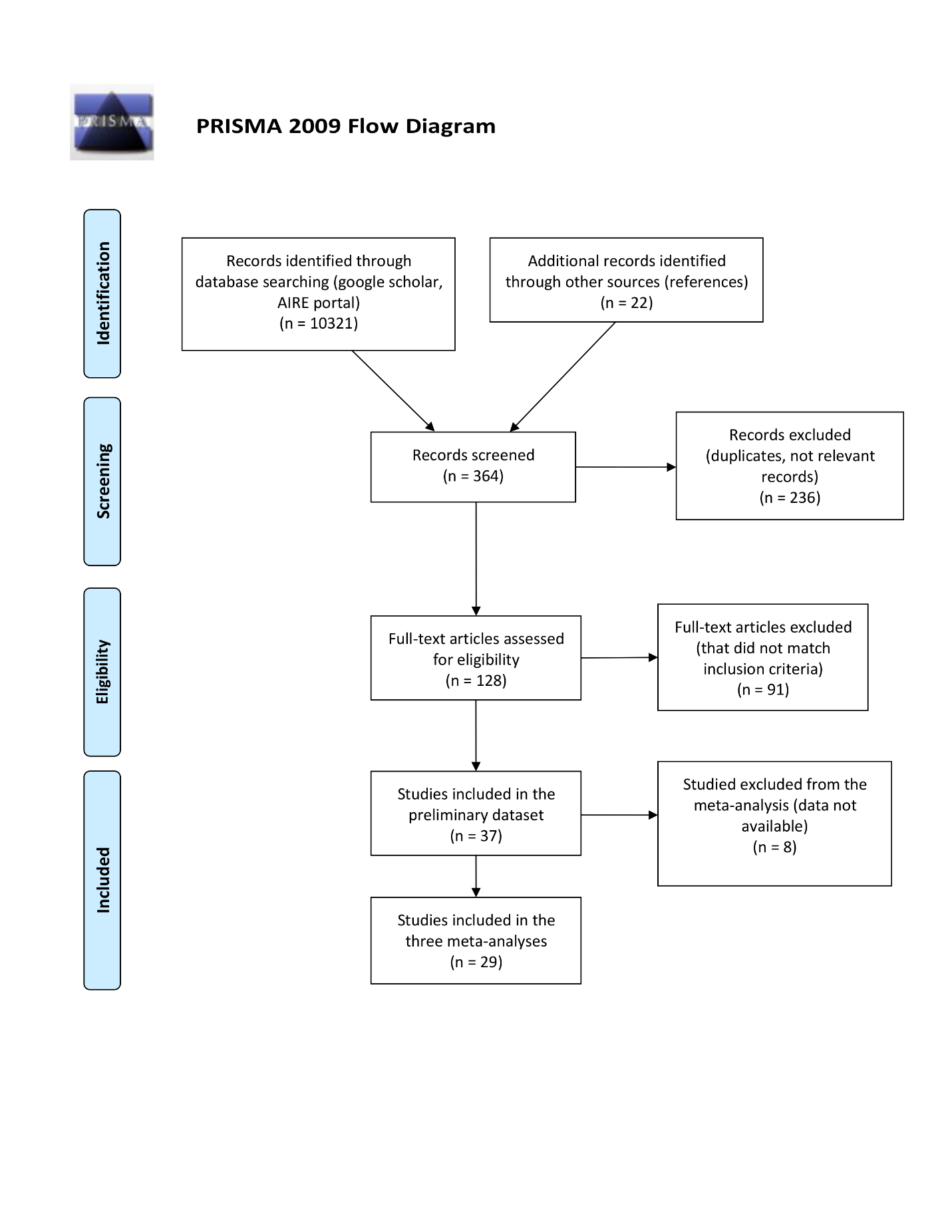

Supplement: S1 Fig — (TIF) [file pone.0191776.s001.tif]
